# Physiotherapy in the ICU: an evidence-based, expert-driven, practical statement

**DOI:** 10.1186/cc14638

**Published:** 2015-03-16

**Authors:** J Sommers, R Engelbert, D Dettling, R Gosselink, P Spronk, J Horn, F Nollet, M Van der Schaaf

**Affiliations:** 1 Academical Medical Center, Amsterdam, the Netherlands; 2 School of Health, Amsterdam, the Netherlands; 3 KU Leuven, Belgium; 4 Gelre Hospital, Apeldoorn, the Netherlands

## Introduction

Evidence-based, expert-driven, practical statements improve quality and effectiveness of the diagnostic and therapeutic process of patient care. Although the effectiveness of physiotherapy treatment strategies in ICU patients has been described, statements or guidelines of physiotherapy for ICU patients are not available [1]. Guidelines on safety management and on the diagnostic and therapeutic process may support and guide clinical decision-making leading towards evidence-based tailored care. The aim of this study was to develop an evidence-based statement for the physiotherapy treatment of ICU patients with recommendations for effective and safe diagnostic assessment and intervention strategies.

## Methods

For the development of this evidence statement, we used the EBRO method, as recommended by the Dutch Evidence Based Guideline Development Platform [2]. This method consists of the identification of clinically relevant research questions, followed by a systematic literature search, quality assessment, and summary of the evidence eventually leading to establishing of concept and final recommendations based on feedback from experts. The final recommendations were prepared according to this methodical approach and summarized in figures, flowcharts and appendices.

## Results

Three expert-based relevant clinical questions were formulated within the physiotherapy clinical reasoning process and were classified according to the International Classification of Functioning, Disability and Health. In a systematic literature search, 129 studies were identified and assessed for methodological quality and classified according to the level of evidence. The final Evidence Statement consisted of recommendations for physiotherapy in ICU patients including safety criteria, a core set of instruments to assess impairments and activity restrictions and effective interventions.

## Conclusion

The Evidence Statement for physiotherapeutic diagnostics and intervention in ICU patients will contribute to the quality of clinical practice by supporting the clinical decision-making process.
